# Performance of Size 1 I-Gel Compared with Size 1 ProSeal Laryngeal Mask in Anesthetized Infants and Neonates

**DOI:** 10.1155/2015/426186

**Published:** 2015-02-22

**Authors:** Gulay Erdogan Kayhan, Zekine Begec, Mukadder Sanli, Ender Gedik, Mahmut Durmus

**Affiliations:** Department of Anesthesiology and Reanimation, Inonu University Medical Faculty, B Blok Daire 11, Malatya, Turkey

## Abstract

*Purpose*. The size 1 I-gel, recommended for small infants and neonates weighing 2–5 kg, has recently been released. There are no prospective studies available that assess the insertion conditions, sealing pressures, or ventilation quality of it. This study was designed to compare the performance of recently released size 1 I-gel with size 1 ProSeal LMA. *Methods*. Fifty infants and neonates, ASA I-II were included in this prospective, randomized, and controlled study. Patients were divided into two groups for placing I-gel or ProSeal LMA. The primary outcome was airway leak pressure, and secondary outcomes included insertion time, insertion success and conditions, initial airway quality, fiberoptic view of the larynx, and complications. *Results*. There were no significant differences in terms of airway leak pressure between the I-gel (27.44 ± 5.67) and ProSeal LMA (23.52 ± 8.15) (*P* = 0.054). The insertion time for the I-gel was shorter (12.6 ± 2.19 s) than for the ProSeal LMA (24.2 ± 6.059 s) (*P* = 0.0001). Insertion success and conditions were similar in groups. We encountered few complications. *Conclusion*. Our study demonstrates that the size 1 I-gel provided an effective and satisfactory airway as the size 1 ProSeal LMA. It may be a good alternative supraglottic airway device for use in small infants and neonates. This trial is registered with: ClinicalTrials.gov NCT01704118.

## 1. Introduction

The I-gel (Intersurgical Ltd., Wokingham, Berkshire, UK) is a disposable supraglottic airway device with noninflatable cuff and is made from thermoplastic elastomer, which is unlike other laryngeal masks [1]. The manufacturer states that I-gel is suitable for hypopharyngeal anatomy, provides good perilaryngeal sealing, and reduces the risk of airway obstruction by preventing intraoral trauma and folding of epiglottis, due to the device's soft and gel-like structure [2]. Studies assessing I-gels of different pediatric sizes, except size 1 (without gastric drainage tube), have shown that the I-gel is an efficient and safe airway device with easy insertion, a high rate of successful insertion, sufficient ventilation, and few complications [1, 3–5]. The size 1 I-gel, recommended for small infants and neonates weighing 2–5 kg, has recently been released for use. There are no prospective studies available that assess the insertion conditions, sealing pressures, or ventilation quality of it.

The ProSeal laryngeal mask (PLMA) (LMA North America, Inc., San Diego, USA) is a modified type of LMA (larger and deeper bowl, enlarged and softer cuff) with a gastric drainage tube. Unlike the PLMA for adults, the size 1 PLMA for infants and neonates below 5 kg has no additional cuffs on its dorsal side similar to other pediatric sizes. There are several studies comparing the size 1 PLMA with size 1 classic LMA and showing its superiority [6, 7].

The objective of this prospective, randomized, and controlled study is to compare the performance of the recently released size 1 I-gel and the size 1 PLMA, which has been proven to be superior to the classical LMA in prospective studies. In this study, the primary endpoint was the airway leak pressure, and the secondary endpoints were the insertion time, insertion success and conditions, initial airway quality, fiberoptic view, and complications.

## 2. Materials and Methods

This study was carried out after obtaining approval from the Local Ethical Committee of the Faculty of Medicine, Inonu University (Acceptance No. 2011/89), Malatya, Turkey (Chairperson Professor M. Genc), on 5 July 2011. Informed consent was obtained from parents of infants and neonates. Fifty infants and neonates, ASA I-II, and weighing 2–5 kg were included into the study. Infants who required a supraglottic airway device and were scheduled for elective surgery under general anesthesia were chosen for the study. Infants who had a history of pulmonary disease, expected to have aspiration (gastroesophageal reflux, gastrointestinal stenosis, or stricture) and a difficult airway, were excluded from the study.

Infants were administered rectal 30 mg/kg paracetamol 1 hour before the operation and taken into operating room after a peripheral venous cannulation and began hourly fluid infusion on the Pediatric Surgery Services. Routine monitoring (ECG, pulse oximetry, blood pressure, and temperature) was performed while the infants were on the operating table that was covered by a heating blanket. The randomization was performed by sealed envelope method. An anesthesiologist who was blinded to the study opened a sealed envelope and prepared the device. Lidocaine 1 mg/kg, remifentanil 1 mcg/kg (slow bolus in approximately 1 min), and 3 mg/kg propofol were administered on anesthesia induction; no muscle relaxant was administered. The infants were ventilated with a facemask until conditions were suitable for laryngeal mask insertion (loss of eyelash reflex, jaw relaxation, and the absence of movement). If sufficient anesthetic depth was not achieved, an additional 1 mg/kg propofol was administered. In Group P, a PLMA with fully deflated cuff and applied water-based lubricant was inserted using a metal introducer. After insertion, the cuff was inflated with the recommended volume of air and the cuff pressure was adjusted to 60 cmH_2_O using a manometer (Endotest pressure manometer, Rüsch, Germany). In Group I, the I-gel with its lubricated cuff was orally inserted along the hard palate until resistance was felt, as recommended by the manufacturer. Afterwards, patients in both groups were connected to the circle system of anesthesia machine and were ventilated manually. Anesthesia was maintained with 4 L/min fresh gases, consisting of 2.5%–3% sevoflurane and 50%/50% O_2_/N_2_O. An experienced and same anesthesiologist performed the insertion procedures in all patients.

The time between picking up the prepared PLMA (with introducer and deflated cuff) or the I-gel and the appearance of the first stable capnographic trace was recorded as the insertion time. As previously described, the conditions for insertion were scored according to mouth opening (1: full; 2: partial; 3: nil), gagging or coughing (1: nil; 2: slight; 3: gross), swallowing (1: nil; 2: slight; 3: gross), head or limb movement (1: nil; 2: slight; 3: gross), laryngospasm (1: nil; 2: slight; 3: complete), and ease of insertion (1: easy; 2: difficult; 3: impossible) [8, 9]. A summed score was obtained by adding the scores collected for each patient. The initial airway quality was evaluated with manual ventilation by adjusting APL valve to 20 cmH_2_O. The evaluation was performed by listening to the lungs, epigastrium, and perilaryngeal field and observing the expansion of thorax. The following scale was used: excellent (no leaks heard), good/acceptable (a slight, clinically insignificant leak and sufficient ventilation), and poor/unacceptable (significant leak and insufficient ventilation which requires reposition or relocation of the device). Next, the airway device was taped, and the head was fixed in a neutral position. In the event of unsuccessful insertion of the device or insufficient ventilation despite two attempts, a muscle relaxant was administered, and endotracheal intubation was performed. The number of attempts required to position the device was recorded.

Fresh gas flow was adjusted to 3 L/min, and after closing the expiratory valve, the airway pressure at which an audible leak in the mouth was heard was recorded as the “*P*_leak_”. If *P*_leak_ reached 35 cmH_2_O, the expiratory valve was opened [6]. After adjusting the APL valve to 35 cmH_2_O, the maximum tidal volume (TV_max⁡_) was measured by squeezing the balloon of anesthesia circuit until an audible leak occurred [6]. The presence of gastric insufflation was detected by auscultation of the stomach.

To evaluate the anatomic position of the supraglottic device, a fiberoptic evaluation was performed. Before fiberoptic evaluation, 1 mcg/kg of additional fentanil was administered to the patients. The breathing system was disconnected and the fiberoptic bronchoscope (11302BD2, diameter 3,7 mm; length 65 cm; Karl Storz, Tuttlingen, Germany) was inserted through the ventilation tube to evaluate the glottic view. Fiberoptic images were recorded using a digital camera and stored on a personal computer for grading by an independent anesthetist. The images were graded with a score from 1 to 5, which has been defined and proposed previously [10, 11] (grade 1-only larynx seen; grade 2-larynx and epiglottis posterior surface seen; grade 3-larynx and epiglottis tip of anterior surface seen, <50% visual obstruction of epiglottis to larynx; grade 4-epiglottis downfolded and its anterior surface seen, >50% visual obstruction of epiglottis to larynx; and grade 5-epiglottis downfolded and larynx cannot be seen directly).

Additionally, the heart rate and mean blood pressure values were recorded. In Group P, a lubricated 8 F gastric tube was inserted through the gastric drainage tube. The position of the gastric tube was verified by air injection and aspiration of gastric contents. After assessments and evaluations, mechanical ventilation using pressure-controlled ventilation was started (peak pressure 10–14 mmHg and respiratory rate 35–40/min to obtain a tidal volume 8–10 mL/kg).

At the end of the operation, wound infiltration was done with 1 mg/kg 0.25% bupivacaine. The inhalation agent was stopped, and the airway device was removed, upon observing sufficient spontaneous ventilation and protective reflexes. Complications encountered during or at the end of the operation, such as desaturation (SpO_2_ less than 90%), gastric insufflations, aspiration, laryngospasm, bronchospasm, and blood stain on the airway device during removal, were recorded.

The null hypothesis was that there was no difference in *P*_leak_ between the size 1 I-gel and the size 1 PLMA. We did a pilot study of 10 patients to detect a difference of 10% between the groups with a standard deviation of 4. Therefore, for a power of 0.8 and *α* = 0.05, a sample size of 24 patients in each group was required. Twenty-five patients in each group were recruited because of the possibility of dropouts. Statistical analyses were performed using SPSS for windows, version 13.0 program. Continuous data were reported as mean ± SD. Categorical data were reported as number and proportion. Normality for continuous variables in groups was determined by the Shapiro Wilk test. The variables showed a normal distribution (*P* > 0.05). Therefore, an unpaired *t* test was used for comparison of variables between the groups. A paired *t* test was used for variables within the groups. Pearson chi-square and Fisher's exact tests were used for the categorical data. A value of *P* < 0.05 was considered to be significant.

## 3. Results

**Figure 1:**
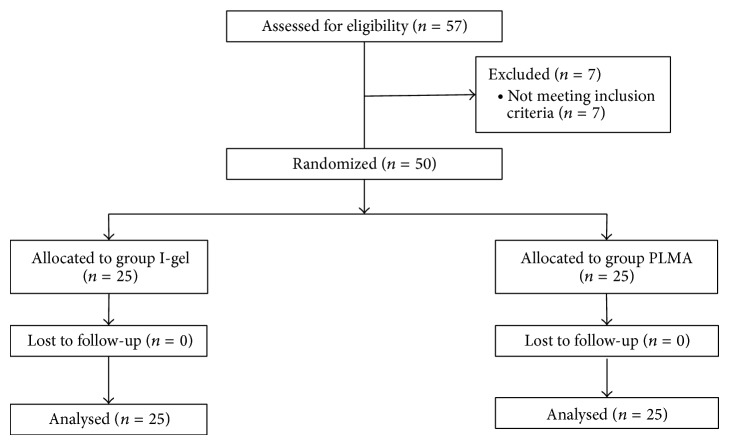


Fifty-seven patients were eligible for the study. Seven of the patients were excluded from the study because they did not meet the inclusion criteria. Fifty patients completed the study, and no patients were excluded from the analysis (Figure 1). There were no significant differences between the two groups in terms of demographic data, ASA status, and the need for additional doses of propofol (Table 1). The types of surgery consisted of 41 unilateral inguinal hernia repairs, 7 bilateral inguinal hernia repairs, 1 rectal biopsy, and 1 cystoscopy.

The mean *P*_leak_ values were 27.44 ± 5.67 cmH_2_O in Group I and 23.52 ± 8.15 cmH_2_O in Group P. There were no statistical differences between the groups (*P* = 0.054) (Table 2). The TV_max⁡_ values were 90 ± 45.91 mL and 69.13 ± 36.79 mL in Group I and in Group P, respectively (*P* = 0.091) (Table 2). The expiratory valves were opened in 6 patients in Group I and 4 patients in Group P when the *P*_leak_ value exceeded 35 cmH_2_O. While insertion time was shorter in Group I (12.6 ± 2.19 sec) than in Group P (24.2 ± 6.059 sec) (*P* = 0.0001), insertion success rates and insertion conditions were similar in both groups. First-attempt insertion success and overall success rates were 92% (23 patients) and 96% (24 patients) in Group I, and 88% (22 patients) and 100% (25 patients) in Group P. In a patient for whom insertion failed in Group I, despite suitable insertion conditions and sufficient initial airway quality, a decrease in the ventilation quality (decrease in capnograph trace, low tidal volume) was observed during the operation. Upon failing to achieve sufficient ventilation on the second attempt, the I-gel was removed, and endotracheal intubation was performed. Initial data were recorded for this patient and therefore included in the results.

The initial airway quality was detected to be better in Group I than in Group P (*P* = 0.006). The fiberoptic view scores are shown in Table 2. There were no statistical differences between groups (*P* = 0.085). Grade 1 (only the larynx) was seen in 12 and 5 patients in Group I and Group P, respectively (*P* = 0.036). The number of patients whose larynx was not seen (grade 5) was 0 in Group I and 5 in Group P (*P* = 0.025). It was recorded that the epiglottis could not be seen in 2 of these 5 patients due to downfolding of the PLMA.

Comparisons between groups showed no statistical differences in heart rates and mean blood pressures. Preoperative desaturation developed in 3 patients. Desaturation was observed in 1 patient in Group I due to laryngospasm developed during fiberoptic evaluation, in 1 patient in Group P due to impairment in ventilation after surgery was started, and in 1 patient in Group P due to laryngospasm after removal of PLMA at the end of the operation. Desaturation was quickly eliminated by manual ventilation with 100% O_2_ and increasing the anesthetic depth in the first and second patient. Again, positive pressure manual ventilation with 100% O_2_ administration was sufficient in the third patient and there was no need to succinylcholine for laryngospasm. Gastric insufflations developed in 1 patient in Group I. In Group P, blood stain was detected on the LMA in 2 patients. No aspiration or bronchospasm was observed in any of the patients (Table 3).

## 4. Discussion

In this prospective study, the size 1 I-gel and the size 1 PLMA exhibited similar performances in terms of *P*_leak_ and TV_max⁡_ values, insertion conditions, insertion success, and larynx visibility. However, the size 1 I-gel has advantages over PLMA in terms of insertion time and initial airway quality.

There are few clinical studies on supraglottic airway devices manufactured for small infants and neonates below 5 kg. In comparative studies of the size 1 PLMA and classic LMA, it was reported that *P*_leak_ was higher, the initial airway quality was better, and there was less gastric insufflations with the PLMA [6, 7, 12, 13]. Because of the proven effectiveness and safety of the PLMA in the infants and neonates, we preferred to compare the I-gel with PLMA only.

It was reported that the I-gel, with its different physical characteristics, is an advantageous device for pediatric patients above 5 kg because of its ease of insertion, sufficient ventilation, and ease of gastric tube insertion [1, 3–5, 14–16]. Hughes et al. [14] compared size 2 I-gel with PLMA and classic LMA, in spontaneously breathing children, and they found that size 2 I-gel was easy to insert and provides higher oropharyngeal sealing pressure (OSP) than the others. The detected values of OSP were 26 ± 2.6 cmH_2_O and 23 ± 1.2 cmH_2_O with the size 2 I-gel and size 2 PLMA, respectively. Their values were similar to our study that obtained a sufficient and acceptable *P*_leak_ value (27.44 cmH_2_O) with the size 1 I-gel. Jagannathan et al. suggested that I-gel had higher *P*_leak_ value than the Supreme device in infants and children. The selected sizes of the airway devices were 1.5, 2, 2.5, and 3 in their study and the median *P*_leak_ value was 20 (18–25) cmH_2_O with the I-gel [15]. In a similar manner, Lee et al. detected the mean *P*_leak_ value of size 1.5, 2, and 2.5 I-gels 22 (20–25) cmH_2_O [17]. In these studies, the researchers did not report each *P*_leak_ measurements for the different sizes used in their study. However, Theiler et al., in their study on children from different age groups weighing 5–50 kg, found the average *P*_leak_ value to be 22 ± 5 cmH_2_O with the I-gel and they obtained a significantly higher *P*_leak_ value (27 cmH_2_O) with the size 1.5 I-gel [5]. Similarly, Beringer et al. found the average *P*_leak_ value to be 20 cmH_2_O with sizes 1.5, 2, and 2.5 I-gels; the value obtained with size 1.5 I-gel was higher (26 cmH_2_O) [3]. Furthermore, in our study, the initial airway quality was better and we obtained a sufficient TV_max⁡_ value with the I-gel. As a result, it can be concluded that the small size (size 1 and 1.5) I-gels are suitable for the anatomical structure of infants and neonates and provide satisfactory airway.

The *P*_leak_ for the size 1 PLMA was higher than the *P*_leak_ found by several clinical studies [18] and lower than others [6, 7]. This was caused by the differences in standardization of anesthetic depth and different measuring techniques, such as the maximum airway pressure allowed during measurement or the amount of fresh gas flow [1, 18]. Additionally, different head-neck positions may affect *P*_leak_ values, as well [19, 20].

In our study, we inserted the size 1 PLMA using an introducer because of more flexibility than other sizes of the PLMAs and because of a small introducer strap, and the absence of an integral bite block, as recommended by the manufacturer. We think that removal of the introducer and inflation of the cuff significantly increases the time required for achieving an effective airway. Although times obtained in various studies may be different, the general conclusion is that the I-gel has a short insertion time and is inserted easily [3, 14, 17]. As in many other studies, the insertion time required with the I-gel in our study was quite short. So, I-gel may be advantageous for emergency airway management in infants.

In our study, the larynx could not be seen in 5 patients in the PLMA group, and it was found that the PLMA was downfolded in 2 patients. *P*_leak_ values (15 and 9 cmH_2_O) and leak volumes in these patients were quite low, and the PLMA was inserted twice in one patient due to failure to maintain sufficient ventilation. It was reported that the PLMA might cause upper airway obstruction in pediatric patients and infants due to supraglottic bulging and laryngeal compression. Thus, pediatric PLMA users were recommended to be awake [20, 21]. Hyperinflation of the LMA cuff may cause airway morbidity by increasing the pressure on perilaryngeal structures. Therefore, routine monitoring of the cuff pressure is recommended in pediatric patients [21]. In studies on pediatric patients, low levels of airway trauma were observed with I-gel [1, 3–5]. In our study, blood stain was detected in 2 patients in the PLMA group while no blood was detected in the I-gel group. Absence of a cuff in the I-gel may, as suggested by Lee et al. [17], be thought of as an advantage to avoid these problems.

Previous researchers have warned that pediatric size I-gels are likely to dislodge and should be taped well [3–5, 14]. Although we had no objective data, we agreed with this opinion based on our clinical observations.

The absence of a gastric drainage tube in the size 1 I-gel may be a disadvantage because it may increase the risk of gastric regurgitation or aspiration. Although no aspiration events were observed in any of the patients, the number of patients in our study is quite low, and we think that more clinical studies with larger patient populations are needed to understand whether the size 1 I-gel provides protection against aspiration.

Our study has a number of limitations. One of these limitations is that our data collection was unblinded with the exception of the fiberoptic evaluation. Second, ventilation quality and the *P*_leak_ values were evaluated only in the beginning of the operation. I-gel may provide better sealing of the perilaryngeal area over time by warming with body temperature, due to its thermoplastic characteristic. Third, an anesthesia specialist who had more experience with the size 1 PLMA carried out the insertion procedures. It may be assumed that this affected the results related to insertion. However, the insertion time was found to be shorter for I-gel in our study, despite this disadvantage. Another limitation is that this study had only power for airway leak pressure, not for the other variables.

In conclusion, our study demonstrates that the size 1 I-gel is an effective supraglottic airway device for use in small infants and neonates. In comparison with size 1 PLMA, I-gel is quicker to insert and provides better initial airway quality. Although we encountered few complications, further studies are needed to determine whether size 1 I-gel is reliable for regurgitation and aspiration.

## Figures and Tables

**Table 1:** Demographic data and additional doses of propofol. Values are mean ± standard deviation or number.

Patients	Group I (*n* = 25)	Group P (*n* = 25)	*P* value
Gender (F/M)	5/20	6/19	1.000
Age (month)	2.1 ± 0.7	2.3 ± 1.1	0.448
Weight (kg)	4.2 ± 0.7	4.4 ± 0.9	0.400
ASA status I/II	22/3	20/5	0.700
Additional propofol (mg)	1.7 ± 2.95	3.08 ± 3.46	0.136

ASA Status: American Society of Anesthesiologists Physical Status.

**Table 2:** Conditions during insertion, quality of initial airway, airway leak pressure, maximum tidal volume, and fiberoptic view of size 1 I-gel and PLMA and complications during surgery and emergence. Values are mean ± standard deviation or number (proportion).

	Group I (*n* = 25)	Group P (*n* = 25)	*P* value
Insertion time (s)	12.6 ± 2.19	24.2 ± 6.05	0.0001^†^
First-attempt insertion success *n* (%)	23 (92%)	22 (88%)	0.609^#^
Overall insertion success	24 (96%)	25 (100%)	
Insertion condition summed score	6.2 ± 0.5	6.4 ± 0.5	0.197^†^
Mouth opening (full/partial/nil)	25/0/0	25/0/0	
Gagging or coughing (nil/slight/gross)	25/0/0	25/0/0	
Swallowing (nil/slight/gross)	24/1/0	24/1/0	
Movement (nil/slight/gross)	24/1/0	19/5/1	
Laryngospasm (nil/partial/complete)	25/0/0	25/0/0	
Ease of insertion (easy/difficult/impossible)	23/1/1	22/3/0	
Quality of initial airway (*n*)			0.006^#^
Excellent	19	9	
Good/acceptable	4	15	
Poor/unacceptable	2	1	
Airway leak pressure (*P*_leak_) (cmH_2_O)	27.44 ± 5.67	23.52 ± 8.15	0.054^†^
Maximum tidal volume (TV_max_) (mL)	90 ± 45.91	69.13 ± 36.79	0.091^†^
Fiberoptic view (1/2/3/4/5^*^)	12/4/2/7/0	5/5/3/7/5	0.085^#^
Grade 1 (only larynx seen)	12 (48%)	5 (20%)	0.036^#^
Grade 5 (epiglottis downfolded and larynx cannot be seen directly)	0 (0%)	5 (20%)	0.025^#^

^†^Unpaired *t* test was used for comparison of variables between two groups.

^#^Pearson chi-square and Fisher's exact test were used for comparison of variables between the categorical data.

^*^(1) Only larynx seen; (2) larynx and epiglottis posterior surface seen; (3) larynx and epiglottis tip of anterior surface seen, 50% visual obstruction of epiglottis to larynx; (4) epiglottis downfolded and its anterior surface seen, 50% visual obstruction of epiglottis to larynx; (5) epiglottis downfolded and larynx cannot be seen directly.

**Table 3:** Complications during surgery and emergence. Values are number (proportion).

	Group I (*n* = 25)	Group P (*n* = 25)	*P* value
Desaturation (SpO_2_ < 90%)	3 (12%)	2 (8%)	1.000
Gastric insufflations	1 (4%)	0 (0%)	1.000
Aspiration	0 (0%)	0 (0%)	—
Laryngospasm	1 (4%)	1 (4%)	1.000
Bronchospasm	0 (0%)	0 (0%)	—
Blood stain	0 (0%)	2 (8%)	0.490
